# Individual differences in sociocognitive traits in semi‐free‐ranging rhesus monkeys (*Macaca mulatta*)

**DOI:** 10.1002/ajp.23660

**Published:** 2024-07-04

**Authors:** Alexis A. Diaz, Raisa Hernández‐Pacheco, Alexandra G. Rosati

**Affiliations:** ^1^ Department of Biology Stanford University Stanford California USA; ^2^ Department of Biological Sciences California State University Long Beach California USA; ^3^ Departments of Psychology and Anthropology University of Michigan Ann Arbor Michigan USA

**Keywords:** comparative development, emotional processing, gaze following, primate cognition

## Abstract

Characterizing individual differences in cognition is crucial for understanding the evolution of cognition as well as to test the biological consequences of different cognitive traits. Here, we harnessed the strengths of a uniquely large, naturally‐living primate population at the Cayo Santiago Biological Field Station to characterized individual differences in rhesus monkey performance across two social cognitive tasks. A total of *n* = 204 semi‐free‐ranging adult rhesus monkeys participated in a data collection procedure, where we aimed to test individuals on both tasks at two time‐points that were one year apart. In the *socioemotional responses task,* we assessed monkeys' attention to conspecific photographs with neutral versus negative emotional expressions. We found that monkeys showed overall declines in interest in conspecific photographs with age, but relative increases in attention to threat stimuli specifically, and further that these responses exhibited long‐term stability across repeated testing. In the *gaze following task* we assessed monkeys' propensity to co‐orient with an experimenter. Here, we found no evidence for age‐related change in responses, and responses showed only limited repeatability over time. Finally, we found some evidence for common individual variation for performance across the tasks: monkeys that showed greater interest in conspecific photographs were more likely to follow a human's gaze. These results show how studies of comparative cognitive development and aging can provide insights into the evolution of cognition, and identify core primate social cognitive traits that may be related across and within individuals.

## Research Highlights

- We examined individual variation and longitudinal patterns of cognitive performance in semi‐free‐ranging rhesus macaques, using two social cognitive tasks
- Monkeys showed distinct age‐related change in a task measuring responses to socioemotional signals, and their performance exhibited long‐term repeatability
- Monkeys did not show clear age shifts in a task measuring gaze following (co‐orienting) responses, and individual performance was not clearly stable over time
- Individual differences approaches can provide new insights into the evolution of cognitive abilities across species

## Graphical abstract

We tested a large sample of monkeys longitudinally on two sociocognitive tasks to examine individual differences in cognitive responses. (a) In the *socioemotional responses* task, we measured preferences for viewing photographs of conspecifics producing emotional versus neutral expressions. (b) In the *gaze following* task, we measured co‐orienting responses with a demonstrator. We found that monkeys showed repeatable responses with clear age‐related differences in the socioemotional responses task, but not in the gaze following task. In addition, monkeys that showed greater interest in conspecific photographs were more likely to follow a human's gaze.

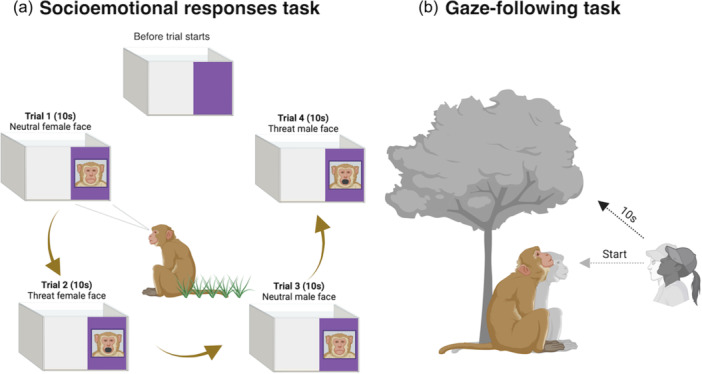

## 1 INTRODUCTION

Comparative studies of cognition in animal species are important for understanding the evolutionary history and biological consequences of different cognitive skills (Pritchard et al., 2016; Rosati et al., 2022; Thornton & Truskanov, 2022). While much work on animal cognition has focused on testing whether different species have a particular skill and in comparing variation in performance across species (Bond et al., 2007; De Petrillo, Bettle, et al., 2022; Herrmann et al., 2007; Maclean et al., 2014), there is increasing appreciation that an individual differences approach to examining intraspecific differences—how and why individuals of the same population or species solve problems—is also crucial for understanding the evolution of cognition (Thornton & Lukas, 2012; Völter et al., 2018). In particular, an individual differences approach aimed at characterizing intraspecific variation in cognition is needed to address several core questions concerning the evolutionary function of cognition and behavior, including whether animals show age‐related change in cognition as they develop; whether sexes differ in their responses to problems; and finally, whether cognitive performance represents a stable and potentially heritable trait.

First, an individual differences approach allows for characterization of age‐related changes in cognition that shape how different individuals respond to important challenges in their environment (Rathke & Fischer, 2020; Rosati et al., 2016; Soravia et al., 2022). It is well‐recognized that human cognition develops and changes over the lifespan, and emerging work on comparative cognitive development aims to compare the ontogeny of cognitive abilities across different species (Gomez, 2005; Matsuzawa et al., 2006; Matsuzawa, 2007; Rosati et al., 2014). Integrating perspectives from both developmental psychology and evolutionary biology is crucial to understand how animals shift their cognition and behavior during different life stages (Almeling et al., 2016; Almeling et al., 2017; Fischer, 2017; Haux et al., 2022; Machanda & Rosati, 2020; Rathke & Fischer, 2020; Rosati et al., 2016; Rosati et al., 2020; Rosati et al., 2023; Rosati, Arre, et al., 2018). For example, species can vary widely in their life history characteristics compared to humans (Alberts et al., 2013), and differences in the pace of growth and length of lifespan has been linked to important differences in brain maturation during the juvenile period and age‐related senescence (Leigh, 2004, 2012). As such, studies of comparative development can test how cognitive shifts across the lifespan enable adaptive behavior at different life history phases as well as how these shifts are connected to physiological changes in the mind and body.

Another important aspect of individual differences in cognition concerns whether there are sex differences in performance. While many species show pervasive sex differences in their adult behavior, surprisingly there are few demonstrations of similar differences in cognition. For example, chimpanzees are an extremely well‐studied primate both in terms of behavior and cognition, and show major differences in patterns of social behavior and foraging that are thought to reflect adaptive differences in how males versus females maximize their reproductive success (Boesch & Boesch, 1981; Gilby et al., 2017; Lonsdorf, 2005; Machanda et al., 2013; Sabbi et al., 2021; Thompson Gonzalez et al., 2021). In particular, males are much more social than females in wild populations (Gilby & Wrangham, 2008; Machanda et al., 2013; Pusey et al., 2007; Wrangham, 2000). One possibility is that these differences in behavior reflect fundamental differences in underlying cognitive mechanisms—for example, males may have more robust social cognition. However, current work suggests few sex differences in a variety of cognitive responses in chimpanzees, including in social cognition (De Petrillo & Rosati, 2021; Herrmann et al., 2010; Wobber et al., 2014) (but see Cantwell et al., 2022; Haux et al., 2022). Yet it is also important to note that many studies of primate cognition are limited by small sample sizes, highlighting the importance of examining large samples of animals to test both sex and age‐related shifts.

Characterizing individual differences is further necessary to identify what components of cognition are stable, potentially heritable, and thus may impact fitness outcomes (Ashton et al., 2018; Bohn et al., 2023; Cauchoix et al., 2018; Croston et al., 2015; Pravosudov, 2022). A primary question here is whether performance on cognitive tasks reflects some stable trait. This is important because stable differences (repeatability) in a behavior sets the upper limit for the heritability of that behavior (Cauchoix et al., 2018; Wilson et al., 2010). Heritability is a crucial prerequisite for natural selection to act on a trait, to and more generally for cognition to have some consistent impact on individual's biological outcomes such as health and longevity. Experimental measures are often assumed to reflect such a stable individual propensity, indexing the skillfulness with which a given individual solve particular problems, yet there is also accumulating evidence that many fleeting contextual factors can influence task performance (Schubiger et al., 2020). For example, animals may differ in their motivation, hunger, or responses to novel aspects of the task (De Petrillo, Bettle, et al., 2022; Herrmann et al., 2011)—all factors that partially reflect transient aspects of their environment as opposed to stable differences. Thus, even though much work in primate cognition has tested animals at a single time point, approaches that assess the repeatability of cognitive performance over time (e.g., Ashton et al., 2018; Bohn et al., 2023; Cauchoix et al., 2018) are important to understand these patterns. While there is increasing evidence for repeatability of behavioral patterns in nonhuman primates (Brent et al., 2014; Tkaczynski et al., 2020), most work to date on repeatability of cognitive performance in animals has examined non‐primates (see Cauchoix et al., 2018 for a metanalysis of studies) (but see Bohn et al., 2023 for evidence from great ape cognition). As such, little is currently known about repeatability of primate cognitive performance.

A final set of questions that can be addressed by an individual differences approach is whether or how performance across different tasks is related. Recently there has been increasing interest in testing the same animals on a larger battery of tasks to characterize this kind of individual variation in cognitive abilities (Shaw & Schmelz, 2017). For example, the 'Primate Cognition Test Battery' comprising multiple cognitive tasks spanning different cognitive domains has now been implanted with numerous primate species including lemurs, monkeys, nonhuman apes, and human children (Fichtel et al., 2020; Herrmann et al., 2007; Herrmann et al., 2010; 2010; Schmitt et al., 2012). Several other studies to date have used two or more tasks to examine interrelationships between different skills (Beran & Hopkins, 2018; De Petrillo, Nair, et al., 2022; Rosati, DiNicola, et al., 2018; Völter et al., 2018; Völter et al., 2022). From a cognitive perspective, this approach can elucidate the underlying structure of cognition, by assessing if performance on different behavioral tasks reflects shared versus divergent underlying cognitive processes supporting performance. Similarly, from a biological perspective this can give new insights into trait covariation across individuals.

In the current study, we used an individual differences approach to examine patterns of social cognition in a large population of semi‐free‐ranging rhesus macaques (*Macaca mulatta*). We focused on social cognitive performance in this particular species for several reasons. First, rhesus monkeys are a highly social primate species that live in large mixed‐sex groups with a variety of social interactions between both kin and non‐kin (Ellis et al., 2019; Rawlins & Kessler, 1986), and as such social cognitive skills are thought to be important for adaptive behavior in this socially‐complex species (Drayton & Santos, 2016; Ghazanfar & Santos, 2004). Second, rhesus share many human‐relevant cognitive features, and in general social cognition is fairly well‐characterized in this species. For example, macaques share human‐like preferences for attending to socially relevant information including gaze direction, emotional expressions, and other's perceptual and goal states (Drayton & Santos, 2016; Drayton & Santos, 2017; Ghazanfar & Santos, 2004; Gothard et al., 2007; Hoffman et al., 2007; Rosati et al., 2016; Rosati, Arre, et al., 2018; Santos et al., 2006). Finally, while rhesus macaques have a fairly long‐life span for a mammal, they also have a shorter lifespan than humans (Alberts et al., 2013; Bogin & Smith, 1996; Leigh, 2004, 2012; Tigges et al., 1988). As such, they may exhibit important differences in patterns of cognitive development and aging compared to humans. Indeed, work to date has revealed a mixed picture concerning shared versus divergent patterns of cognitive development, with shared patterns in some contexts but not others (Hernández‐Pacheco et al., 2023; Newman et al., 2023).

We specifically focused on two distinct aspects of social cognition in rhesus macaques. First, we tested animals' responses to socioemotional signals by measuring their interest and attention to conspecific photographs. In humans, there are well‐documented changes in socioemotional processing across the lifespan. For example, whereas children and younger adults exhibit a “negativity” bias, selectively attending to and remembering negative stimuli such as negative emotional faces, older adults rather exhibit a positivity bias showing less interest in such faces (Carstensen & Mikels, 2005; Carstensen et al., 2011; Mather & Carstensen, 2005). Understanding these kinds of shifts during aging has also emerged as an important target of comparative research on other animals (Almeling et al., 2016; Almeling et al., 2017; Fischer, 2017; Machanda & Rosati, 2020; Rosati et al., 2020; Thompson González et al., 2021). In particular, experimental work with a cross‐sectional sample of rhesus monkeys ranging from juvenility to old age compared their duration of looking towards photos of negative conspecific signals (threat displays) versus matched neutral expressions. This revealed that while monkeys show clear age‐related shifts in responses, it was actually the opposite pattern of that observed in humans: older monkeys were more attentive to negative emotional stimuli than were younger monkeys (Rosati, Arre, et al., 2018).

Second, we tested monkeys' abilities to gaze follow, or co‐orient with others. In humans, gaze following is a foundational social skill that emerges early in infancy (D'Entremont, Hains, & Muir, 1997), and is thought to be a crucial building block for later‐emerging social cognitive skills, including both communication and components of theory of mind such as inferring other's perceptions (Bettle & Rosati, 2021; Brooks & Meltzoff, 2005; Emery, 2000; Shepherd, 2010; Wellman, 2011). Gaze following is also a good target for studies of comparative development as it shows characteristic shifts across the lifespan in humans: adult women exhibit greater responsivity to gaze cues than do men (Alwall et al., 2010; Bayliss et al., 2005; Deaner et al., 2007; Mundy et al., 2007), and older adults show declines in responsivity to gaze cues (Kuhn et al., 2015; Slessor et al., 2008; Slessor et al., 2016). Many nonhuman animals also follow gaze to some degree (Rosati & Hare, 2009; Shepherd, 2010), and there is emerging evidence concerning the developmental trajectory of this skill across species: some primate species appear to develop gaze following skills much more slowly than humans (Ferrari et al., 2000; Ferrari et al., 2008; Okamoto et al., 2002; Okamoto‐Barth et al., 2008; Teufel et al., 2010; Tomasello & Carpenter, 2005; Tomasello et al., 2001; Tomonaga et al., 2004; Wobber et al., 2014). Conversely, prior work with rhesus monkeys specifically suggests they show a pattern mirroring humans, with this skill emerging in infancy and then declining in old age (Rosati et al., 2016; Tomasello et al., 2001).

In the current study, we adapted methods from prior work examining socioemotional biases (Rosati, Arre, et al., 2018) and gaze following (Rosati et al., 2016) in the Cayo Santiago rhesus macaques using pre‐registered data collection methods. While those prior studies used a cross‐sectional approach testing monkeys from juvenility to old age, here we rather used these validated tasks to collect systematic longitudinal data from a large sample of monkeys on both tasks. Specifically, we tested each monkey on both tasks at two different time points, approximately one year apart, to capture long‐term patterns in cognitive performance. In addition, our sample here consisted of primarily adults to focus on individual variation and age‐related shifts during the adult lifespan. This approach allowed us to test several interrelated questions about individual differences in cognition: (1) whether individuals show any consistent age‐ or sex‐related differences in cognitive responses; (2) whether performance in tasks reflects traits that are stable over time; and (3) whether an individual's performance across these different sociocognitive metrics is related.

## 2 METHODS

**Figure 1:**
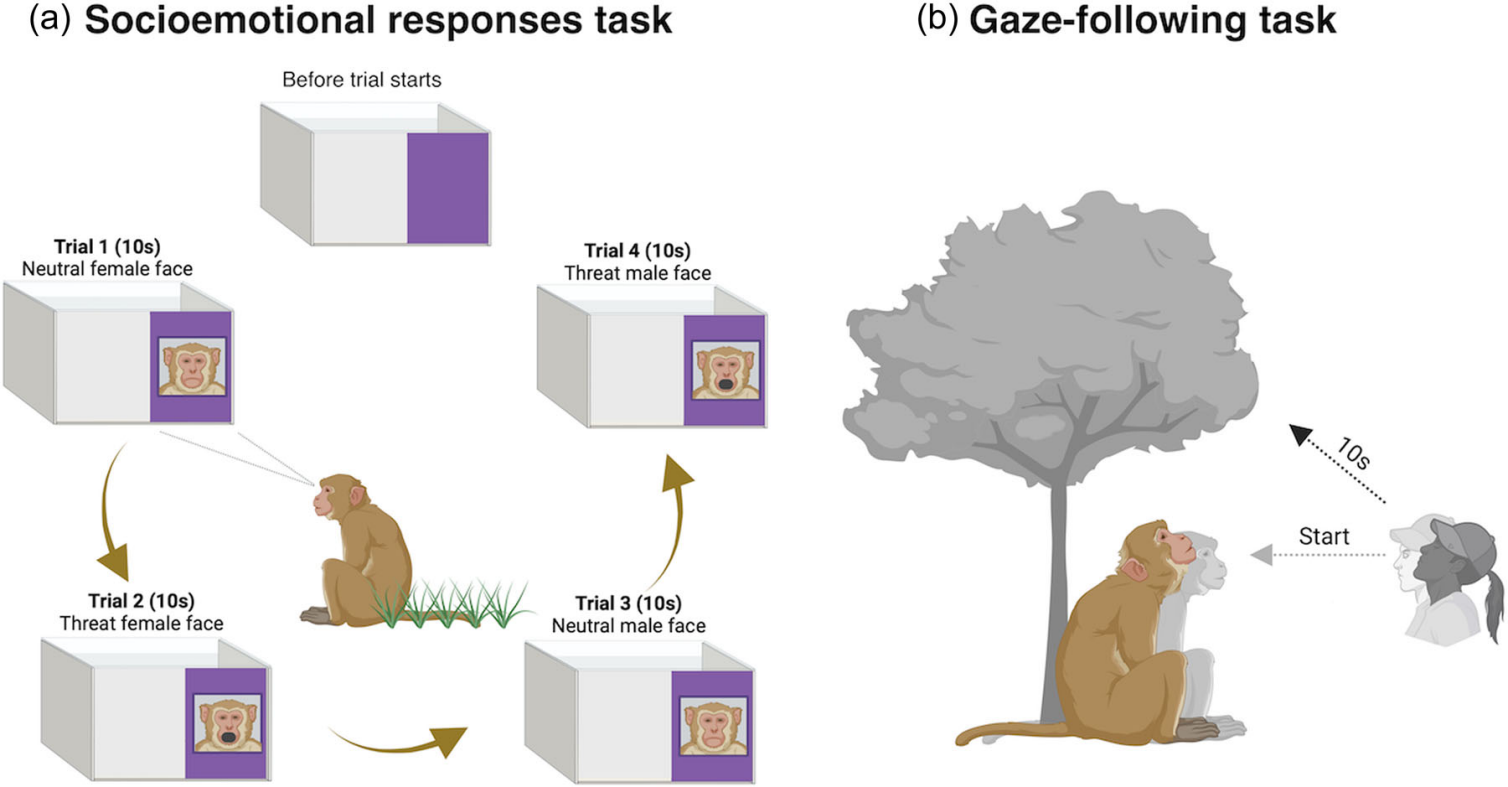
Experimental setup for the sociocognitive tasks. (a) The *socioemotional responses* task measured preferences for viewing photographs of conspecifics producing emotional versus neutral expressions. Monkeys were presented with up to four photos, each for 10 s: a neutral female face (trial 1), followed by a threat face of the same female (trial 2), and a neutral male face (trial 3), followed by a threat face of the same male (trial 4). Before each trial the apparatus had a purple cover over the window, and the experimenter would remove the cover so the monkey could see the photo to start each trial. (b) The *gaze following* task measured co‐orienting responses with a demonstrator. Monkeys completed up to four trials where the experimenter captured the monkey's attention and then looked directly upward for 10 s; we measured whether the monkey also looked up during this period.

In our pre‐registered data collection procedure (https://aspredicted.org/48K_8XP), we tested a sample of adult monkeys from the Cayo Santiago Biological Field Station on two sociocognitive tasks (see Figure 1). The *socioemotional responses task* measured preferences for viewing photographs of conspecifics producing emotional versus neutral expressions, and the *gaze following task* measured co‐orienting responses with a demonstrator. As feasible, individual animals were tested in both tasks at two timepoints, approximately one year apart.

### 2.1 Subjects

Our subjects were rhesus monkeys from the Cayo Santiago Biological Field Station in Puerto Rico. The Cayo Santiago population consists of over 1500 individually identifiable monkeys living in multiple naturally formed social groups on a 38 acre island off the coast of Puerto Rico (Rawlins & Kessler, 1986). Animals are well‐habituated to human observers and are identifiable based on a combination of unique tattoos (three‐character codes), ear notches, as well as aspects of physical appearance. Many monkeys have participated in prior cognitive studies, including tasks involving similar procedures as the current work (Bettle & Rosati, 2019; Dubuc et al., 2016; Higham et al., 2011; Rosati et al., 2016; Rosati, Arre, et al., 2018). For the purposes of our study, we had a target set of primarily adult animals as we were interested in variation in the adult lifespan, rather than early life development. We also focused on monkeys living in two of the groups at Cayo Santiago (F and V) to integrate this cognitive data with other information collected on animals in those groups, such as aspects of their dominance rank and social behavior (these analyses are not part of the current work).

We tested a final sample of 204 monkeys that completed at least one task across the two years of testing. This included 101 females and 103 males, with a mean age of 12.0 years (range: 4.7–29.7 years). In this free‐ranging context, not all individuals could be successfully tested in both tasks in both years (see Table 1 for breakdown of participation across tasks and years). Across analyses, we therefore use different subsets of these monkeys as relevant to test different questions.

**Table 1:** Participation by task and year.

*Year of Test*	*Socioemotional task*	*Gaze Task*	*Both tasks*
*Year 1*	170 (84 F, 86 M)	169 (89 F, 80 M)	143 (74 F, 69 M)
*Year 2*	161 (79 F, 82 M)	108 (55 F, 53 M)	93 (48 F, 45 M)
*Both years*	150 (75 F, 75 M)	102 (55 F, 47 M)	**85 (46** **F, 39** **M)**
*At least one*	181 (88 F, 93 M)	175 (89 F, 86 M)	
*Full sample*	**204 (101** **F, 103** **M)**		

*Note*: Breakdown shows total number of monkey and sex distribution (F = female, M = male) for the two tasks by year. We aimed to test all monkeys in both tasks at both timepoints but in some cases animals were not available (e.g., had died) or could not be located in appropriate testing locations (e.g., where we could place our apparatus). Note that socioemotional task participation here focuses on completion of the first two trials (with female conspecific photographs); some animals completed those trials but did not complete the final two trials (with male photographs) and so are included in only relevant analyses based on participation.

### 2.2 Field experimental methods overview

In the general testing procedure for both tasks, two experimenters walked around the island searching for target monkeys that were sitting calmly and located in an appropriate position for the experimenters to test them (e.g., in a location where we could set the apparatus for the socioemotional task, and near an appropriate tree for the gaze task as noted below). On a given day, each monkey would complete one session of one task; assuming they were located again, they would complete the second task at a later point, typically on a different day when they were similarly located in an appropriate testing position. For monkeys that completed both tasks in a given year, these tests occurred within an approximately two‐month window of testing. Because of the nature of cognitive testing procedures in this free‐ranging context, we did not systematically approach animals for the two tests in any particular order, but rather tested them as feasible when they were located and individually identified depending on which study equipment we had at the time. Then one year later, we conducted the same tests again, attempting to locate the same set of target monkeys within the two‐month testing window.

In a given testing session, experimenter one, the demonstrator, positioned herself approximately 2–3 m away from the monkey. In the socioemotional responses task, she knelt in this location and placed an apparatus on the ground in front of her, so that the monkey could observe successive photos during the task (see Figure 1a and details below). In the gaze following task, she stood in front of the monkey from this distance, and produced gaze cues (see Figure 1b and details below). During both tasks, experimenter two knelt or stood behind the presenter, and filmed the monkey's face with a camera so that their responses could be coded from video.

### 2.3 Socioemotional responses task procedures

This task aimed to characterize individual variation in responses to conspecific photographs that varied in expression (neutral *vs.* threat faces), using procedures following those previously implemented with this population (Rosati, Arre, et al., 2018). As in that prior work, monkeys could view a sequence of photos of unfamiliar conspecifics producing neutral or emotional facial expression, and we measured their looking time to each photo to index relative interest and motivation to view these various stimuli. The first set of two trials showed photos of a female producing a neutral expression, followed by the same female producing a threat expression. The second set of two trials showed a male producing a neutral expression followed by the same male producing a threat expression.

The goal of this study was to assess individual variation in responses, and looking time tasks often produce habituation‐related declined in looking over trials, so following prior work we presented the stimuli in a fixed order (Rosati, Arre, et al., 2018). The logic of this experimental design is that, while this fixed order might not be appropriate to assess group‐level differentiation of the emotional conditions, it is a more sensitive design to detect relative variation in responses across individuals—without additional variation being introduced by the fact that different monkeys viewed the stimuli in different sequences. As in prior work, monkeys could complete up to four trials, and needed to complete at least the first two trials (involving female photographs) to be included in analyses. Some monkeys completed only the female photo trials and were therefore not included in the analyses focusing on responses to male photos. As our prior study showed that monkeys often had quite distinct responses to the female photographs (presented in trials 1–2) and the male photographs (presented in trials 3–4), many of our analyses examined these trials separately.

In test sessions, experimenter one presented the photo stimuli to the monkey using a portable apparatus, while experimenter two filmed the monkey's responses. Specifically, conspecific photos were presented using a white posterboard box (34 cm high, 16 cm deep, 54 cm wide) with a front‐facing window to display the photos (23 cm wide, 30 cm tall). This window was initially covered by a purple flap attached with velcro to the main box. When initiating a trial, experimenter one would rapidly remove the purple cover such that the monkey could observe a photo (see Figure 1a for a diagram of the setup; Supporting Information S1: Figure S1 for photographs of the apparatus and example conspecific stimuli; and see Supporting Information S1: Video S1 for video of the experimental demonstration and example monkey looking responses in the t). Between trials, experimenter one replaced the flap and exchanged the photos out of the monkey's view, and then attracted the monkey's attention to start the next trial as soon as feasible.

On each trial, experimenter one first attracted the monkey's attention to the apparatus by tapping it and calling to the monkey. Once the monkey oriented towards the window, she removed the cover flap to reveal the photo underneath, while simultaneously saying “now;” this auditory cue was used to mark the start of the trial on the video. During each 10 s trial, experimenter one held still after initiating the trial, and gazed downwards so her eyes were occluded by a hat. During this time, experimenter two recorded the monkey's face while timing each 10 s trial on the camera (see Supporting Information S1: Video S1 for an example monkey looking response). After 10 s were up, experimenter two called “stop” to mark the end of the trial on the video recording.

We presented each subject with a series of photos of conspecifics from the Cayo Santiago population showing another monkey's face and upper shoulders. As in prior work, there were two possible photo sets, each comprising an adult female and an adult male (the age of the male and female individuals approximately matched across the two sets). These two sets allowed us to (mostly) match subject monkeys with a set of unfamiliar conspecifics in the photosets. To do so, we aimed to avoide testing subjects with photosets where the subject was living in the photo monkey's natal or current group. The female photos and one of the male photos were the same as in prior work (Rosati, Arre, et al., 2018), but we changed one male set to optimize use of outgroup photos in the current sample. In some cases, however, it was not possible to match a subject with a photoset that did not share either their birth group or current group (or the identity of the monkey was only confirmed after the test was complete). In these cases, we aimed to present photos from individuals who were not current group members, and further controlled for whether outgroup photos were presented in our statistical models.

### 2.4 Gaze following task procedure

This task aimed to characterize individual variation in gaze sensitivity, using procedures that were largely identical to one previously implemented with this population (Rosati et al., 2016). Experimenter one, the demonstrator, first attracted the monkey's attention to her face (by calling to the monkey or snapping her fingers). Once the monkey was judged to be looking, she then looked directly up while simultaneously saying ‘now’ (see Figure 1b for a diagram of the setup; and Supporting Information S1: Video S2 e.g. experimental demonstrations and monkey responses in the task). As in the socioemotional responses task, this auditory cue was used to mark the start of the trial on the video. Experimenter two, the camera person, stood next to experimenter one and filmed the monkey's face. After 10 s trial had concluded, experimenter two said “stop” to mark the end of the trial on the video. We presented monkeys with up to four identical trials. Across the four trials, experimenter one paused for at least 30 s between trials; during this time, she stood looking away from the monkey. This was a change from prior work at Cayo Santiago without such an intertrial delay (e.g., Rosati et al., 2016), and was aimed at reducing habituation across the four trials, a phenomenon where animals who have flexible control over their responses tend to reduce responding to repeated looks (Rosati et al., 2016; Tomasello et al., 2001). Monkeys had to complete at least the first two trials to be included in the final data set.

We used a human demonstrator in this task to ensure the demonstrator's behavior was tightly controlled and consistent. As in prior similar work, there was not a specific target of the experimenter's gaze in this context, so we tested monkeys sitting in the vicinity of a tree such that the human's actions were consistent with the possibility of a target of attention being present, as in prior work using similar gaze following methods (Bettle & Rosati, 2019; Rosati et al., 2016). We therefore did not initiate tests when we observed that another monkey was actually present in a tree above the subject, to avoid any possible visual and auditory confounds.

### 2.5 Exclusions

In this free‐ranging context, monkeys were necessarily tested when other monkeys were also present in the vicinity. As such, some monkeys were approached for testing but would fail to produce a scoreable response, typically because they walked away from the testing area before completing both trials, because they were displaced by other monkeys before completing the session, or because the video was not possible to score (such as due to backlighting, or monkeys moving out of the shot). These sessions did not have scorable responses because the monkeys did not complete the number of trials that was necessary for the study. If the monkey left the testing area or was interfered with while the session was ongoing, experimenter one would judge whether or not it was possible to continue to test the monkey on the subsequent trials based on their spatial position, attention to the apparatus, and social context, following general procedures for field experimental studies at Cayo Santiago (see also Huang et al., 2024). For example, in many cases monkeys may have moved to a location where it was not possible to place the apparatus in an appropriate configuration to complete additional trials in the socioemotional task, or the monkey may have moved away from the tree in the gaze task. Note that experimenter one could make this decision blind to the monkey's specific looking responses in the prior trials, because she was not looking at the monkey the timed 10 s trial in either task.

Numbers of exclusions (*n* = 17 and *n* = 10 monkeys in the socioemotional responses task year one and two, respectively; *n* = 6 and *n* = 5 monkeys in the gaze following task year one and two, respectively) were generally similar to or less than exclusion rates in comparable prior studies in this free ranging population (Bettle & Rosati, 2019, 2021; Hughes & Santos, 2012; Marticorena et al., 2011; Martin & Santos, 2014; Rosati et al., 2016; Rosati, Arre, et al., 2018). In addition, in some cases target animals were approached for testing more than once, because we only confirmed their ID after the test was complete. In this situation, we only included and analyzed their first successful session in the final data set.

### 2.6 Coding

Two coders who were both blind to trial type and condition independently scored all trials from the final set of subjects, resulting in both a primary code and a reliability code. Coders were blind to condition because each individual trial was clipped from longer video sessions, randomized across all trials, and renamed with a random number (e.g., clips 1, 2, 3, etc.), and different trials from the same monkey were interspersed across the full set of clips with no identifying cues. A given trial clip started 1–2 s before the experimenter one said “now” while she was initially attracting the monkey's attention, which allows coders to better judge where the monkey is looking at the start of the trial as a reference, and ended after experimenter two said “stop” (see Supporting Information S1: Video S1 for clips showing monkey looking responses in the socioemotional responses task, and Supporting Information S1: Video S2 for clips showing monkey responses in the gaze following task).

The coders examined these video clips frame‐by‐frame using the program Filmora to assess the monkeys' responses, following typical methods used in prior studies in this population. For looking time responses, we followed the same coding procedures described in prior looking time studies with the Cayo Santiago population (Bettle & Rosati, 2021; De Petrillo & Rosati, 2019; Huang et al., 2024; Hughes & Santos, 2012; Marticorena et al., 2011; Martin & Santos, 2014). Here, the monkey's initial looking direction at the start of the trial (when experimenter one said 'now') served as the reference to code their looking towards the apparatus for the subsequent 10 s. Clips were always coded for 10 s from when experimenter one initiated the trial to equate total trial duration across monkeys. For the gaze following task, we followed the coding procedures of prior studies using this kind of setup with the Cayo Santiago population (Bettle & Rosati, 2019; Bettle & Rosati, 2022; Rosati & Santos, 2017; Rosati et al., 2016). This coding method was similar to those for looking time data, but here the initial looking direction was to the demonstrator, and we rather judged whether the monkey ever looked upwards (using either their eyes or entire head) as our primary measure.

All coders had previous experience coding monkey data by completing a relevant coding training set to be able to reliably code looking time and/or gaze following tasks in this population. AD was the primary coder for all tasks (in both years); there were different secondary reliability coders for each task in each year (this was different people due to coder availability over time). We calculated reliability for our primary measures in each task in each year to check consistency of the codes. In the socioemotional biases task, coders had high correlation in looking times for both year one (Pearson's *r* = 0.93), and year two (Pearson's *r* = 0.97). For the gaze following task, the coders also had high reliability for whether the monkey ever looked up on each trial (Year one Cohen's *κ* = 0.98; Year two Cohen's *κ* = 0.95), as well as the total time they spent looking up in the trial (Year one Pearson's *r* = 0.95; Year two Pearson's *r* = 0.96).

### 2.7 Statistical analyses

Our first goal was to assess patterns of variation in social cognition by age and sex. To address this, we analyzed individual variation in performance in each of the cognitive tasks using multi‐level models, as in our preregistered analysis plan (https://aspredicted.org/48K_8XP; additional aspects of the pre‐registered analysis plan for the entire project focus on associations between cognitive health and social behavior metrics, which are not the focus of the current paper). We used the statistical software program R (R. Core Team, 2022), and implemented mixed models in the *lme4* package (Bates et al., 2015; Bates, 2010). We report Wald's confidence intervals in parameter reporting using the function *confint* from that package. Across tasks, we used likelihood ratio tests (Bolker et al., 2009) to first compare base versus full model fit, and then tested the sequential inclusion of specific predictor variables of interest. We conducted post hoc Tukey comparisons of model factors using the *emmeans* package (Lenth, 2018). We checked that models met assumptions (homogeneity and normality of residuals) using the *DHARMa* package in R (Hartig, 2022).

Depending on the structure of the cognitive data, we either used the *glmer* function to implement generalized linear mixed‐effects models (GLMMs), or the *lmer* function to implement linear mixed‐effects models (LMMs). In the socioemotional responses task, initial checks showed that looking time data was fairly right skewed, so we used GLMMs implemented with a log‐link gamma distribution as is recommended for such data (Lo & Andrews, 2015). Such a modeling approach is similar to log‐transforming the data but rather implements generalize models that account for the right‐skew. For analyses of difference scores in this task (see details of this score's calculation below), we used LMMs because the distribution was fairly normal. In the gaze task, the primary measure was a binary outcome, so we implemented GLMMs with a binomial distribution. In some secondary analyses we used duration of looking as a proxy for co‐orienting responses; we used gamma GLMMs to model this data as it was also right‐skewed. Some models examining looking time responses still did not meet all model assumptions even when accounting for right‐skew in this way, but we found largely similar results implementing these tests with a different structure, (e.g., using LMMs) suggesting these patterns were generally robust to the model structure choices.

For our second goal of assessing performance over time and across tasks, we completed several additional analyses. First, we examined individuals who completed the same task at both time points, using the *rptR* package in R to quantify long‐term repeatability in performance. For a given task, we estimated repeatability after accounting for age and sex for each trial separately (note that these trials measured distinct aspects of responses, and that inclusion of multiple trials from the same task would rather measure short‐term repeatability within the same testing session). Here, we used LMMs for the socioemotional responses as this was the most appropriate option implemented in this package, and binomial GLMMs for the gaze following task. Second, we further used mixed models to test if the first year's cognitive responses were a significant predictor of the second year's responses in the task, using the same model structure as for the main task assessments. This allowed us to address stability in responses over time using a converging approach where multiple trials could be assessed holistically.

Then, we examined within‐individual (longitudinal) age‐related changes in cognitive performance. To do so, we estimated repeated measures correlations implemented with the R package *rmcorr*, which tests for a common within‐individual association for paired measures taken at two or more time points for multiple subjects (Bakdash & Marusich, 2017). Repeated measures correlation is a method that is close to a null multilevel model of varying intercept and a common slope for each individual, but specifically tests for the association shared among individuals (i.e., common regression slope), after adjusting for interindividual variability (Bakdash & Marusich, 2017). We used this to examine within‐subject (longitudinal) change in looking times with age, as our primary mixed model analyses incorporate both longitudinal and cross‐sectional age‐related changes. For each trial, we first used repeated measures correlations to isolate the common intra‐individual variance in looking time across age reflecting longitudinal changes. We further constructed a LMMs with random intercepts for individual identity to estimate age effects in the same sample. This allowed us to report quantitative estimates of both overall age‐related change (incorporating both longitudinal within‐subjects and cross‐sectional between‐subjects age shifts), to compare with the common longitudinal change estimated from the repeated measures correlations.

Finally, for our third goal we assessed if performance across the two different social tasks were related to evaluate if these tasks recruited overlapping versus distinct cognitive processes. For this, we examined the subset of individuals who completed both tasks in the same year and used performance in one task to predict performance in the other. Since animals may have only completed the first two trials in each task, when that task was used as a predictor variable for the other task, we averaged just the first two trials that all animals had completed to make this predictor equitable across individuals. We then used GLMMs to predict responding in one task based on performance in the other, using the same approach as the primary modeling of tasks responses but then adding performance in the other task to test if it further improved model fit.

We analyzed these data with age in years as a continuous predictor, but in some figures we split individuals into age cohorts: younger monkeys up to 15 years; and older monkeys over 15 years, as monkeys in this population have a median lifespan of 15 years and rarely exceed 25 years (Hoffman et al., 2010). While prior work also included infant and/or juvenile cohorts comprising individuals under the age of 5 years (Rosati et al., 2016; Rosati, Arre, et al., 2018), we tested very few monkeys between the ages of 4–5 years in the current study and included those individuals in the younger adult cohort for visualization purposes.

## 3 RESULTS

### 3.1 Individual variation in the socioemotional responses task

**Figure 2:**
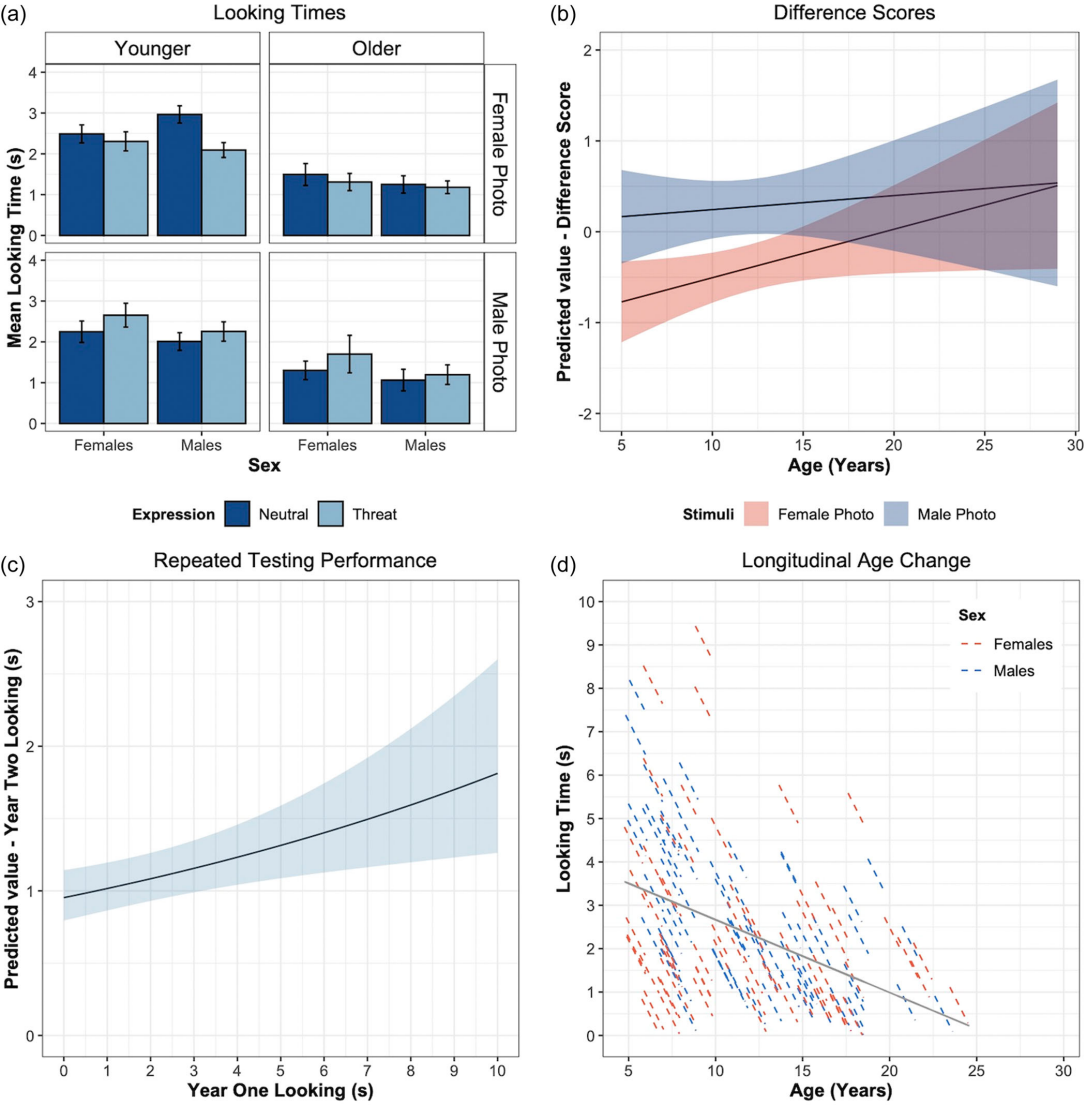
Individual variation in the socioemotional responses task. (a) Mean looking time to conspecific neutral and threat photographs. Graph shows mean duration of looking time towards neutral versus threat stimuli for male and female photographs, broken down by subjects' age cohort and sex. Error bars indicate standard error. (b) Predicted changes in difference scores (negativity bias) with age. Each subject was assigned a difference score indexing their negativity bias (threat looking time–neutral looking time) for female and male photographs, which increased with age. Estimated values are derived from mixed models also accounting for subjects' identity, sex, age, and experimental variables. Ribbons indicate 95% confidence intervals. (c) Predicted performance over repeated testing. We estimated how performance in year two of testing was related to year one performance, for individuals who completed both years of testing. Estimated values are derived from mixed models also accounting for subjects' identity, sex, age, and experimental variables. Ribbons indicate 95% confidence intervals. (d) Longitudinal changes in looking responses. We used repeated measures correlations to isolate within‐individual associations in looking time. The panel depicts responses to the first trial (female neutral photos). The solid gray line indicates the overall linear relationship between age and looking time in the data set incorporating both within‐ and between‐individual age effects (estimated from a mixed model), whereas the dashed lines indicate the common within‐individual age‐related change estimated from repeated measures correlation.

We first examined duration of looking to the photo stimuli across the 2 years of data. There were 181 total monkeys in the sample (all of them completed at least the first two trials presenting female photos in at least one year of testing; see Table 1 for breakdown of participation across years). Overall, monkeys looked an average of M = 2.35 ± SE = 0.13 s at the female neutral photo; 1.93 ± 0.11 s at the female threat photo; 1.89 ± 0.14 s at the male neutral photo; and 2.19 ± 0.15 s at the male threat photo (see Figure 2a for breakdown by subject's sex and age cohort). In an initial analysis of the full data set, we implemented GLMM models with gamma‐log link distribution. The base model accounted for *photo set* (which of the two sets of stimuli was presented), *outgroup photos* (whether monkeys saw unfamiliar conspecifics as the female and male stimuli), and subject's *identity* as a random intercept. We then compared this to a full model including (1) the interaction between *face stimuli (*female *vs.* male photos) X *expression* (neutral *vs.* threat photos), the key index of the different kinds of stimuli presented in the task; (2) subject's *age*; and (3) subject's *sex* to test if these primary variables of interest predicted response. Indeed, this improved fit [χ2 = 68.79, df = 5, *p* < 0.0001]. We then checked the sequential addition of these variables. There was significant improvement with the inclusion of the *face X expression* interaction, the key index of the different stimuli presented, compared to the base model [χ2 = 24.96, df = 3, *p* < 0.0001]. Post hoc tests indicated that monkeys generally looked longer at the female photos than the male photos [*p* < 0.005], which is not surprising since they were presented first in the sequence, but also that while monkeys looked significantly less at the female threat photo compared to the female neutral photo [*p* < 0.0001], they showed no such decline for the male threat photo compared to the male neutral photo [*p* = 0.16, n.s.]. The inclusion of *age* also improved fit [χ2 = 43.83, df = 1, *p* < 0.0001]: monkeys looked less at photographs overall as they aged. Further inclusion of subject's *sex* did not improve fit [χ2 = 0.004, df = 1, *p* = 0.95, n.s.], indicating no differences between the responses of male and female monkeys. Overall, this initial analysis indicated that monkeys show age‐related shifts in looking during adulthood, but also qualitatively different reactions to the female versus male emotional stimuli.

Given that these different patterns of responding to the female versus male stimuli mirrored those seen in prior work, and that distinct subsets of monkeys completed either just the first two female stimuli trials versus the male stimuli trials as well, we then examined more nuanced aspects of these age‐related shifts in separate analyses of responses to the female and male stimuli, using the same analytic approach from prior work with this task (Rosati, Arre, et al., 2018). To examine responses to the female stimuli, we implemented a base GLMM model accounting for *photo set* (which of the two sets of stimuli was presented), *outgroup photos* (whether monkeys saw unfamiliar conspecifics as the female stimuli), and subject's *identity* as a random intercept. We then compared this to a full model including (1) photo e*xpression* (neutral *vs.* threat); (2) subject's *age;* (3) subject's *sex*; and (4) an *expression X age* interaction, the main test of whether socioemotional biases change across the lifespan*.* The full model showed improved model fit compared to the base model [χ2 = 67.51, df = 4, *p* < 0.0001]. We then checked the sequential addition of these variables. First, there was significant improvement with the inclusion of *expression* compared to the base model [χ2 = 15.08, df = 1, *p* = 0.001]: monkeys viewed the female neutral photo longer than the threat photo. Adding in *age* further improved fit [χ2 = 47.15, df = 1, *p* < 0.0001]: overall, monkeys looked less at photos with increasing age. In contrast, there was no improvement with inclusion of *sex* [χ2 = 0.83, df = 1, *p* = 0.36, n.s.], indicating no differences in responses between males and females. Finally, the *expression X age* interaction further improved fit [χ2 = 4.45, df = 1, *p* = 0.034; see supplement for model parameters]. Post‐hoc tests revealed a significant difference in age slopes for responses to the neutral versus threat photo [*p* = 0.034], such that older monkeys exhibited an attenuated a decline in looking at the threat compared to the neutral photo. Additional checks confirmed that these patterns held for monkeys tested with both photo sets, with no differences according to the photo set that was presented.

We used the same approach to examine looking to the male photo stimuli. As for female photos, the inclusion of all experimental variables improved fit in the full model compared to the base model [χ2 = 18.29, df = 4, *p* = 0.001]. We then checked the sequential addition of these variables. Here, inclusion of *expression* did not improve model fit compared to the base model, unlike with the female photos [χ2 = 1.52, df = 1, *p* = 0.22, n.s.]. Further inclusion of *age* did improve fit compared to the base model [χ2 = 16.96, df = 2, *p* = 0.0002]: overall, monkeys looked less at photos with increasing age. There was again no improvement with inclusion of *sex* [χ2 = 0.54, df = 1, *p* = 0.46, n.s.], indicating no differences between males and females. We finally added the *expression X age* interaction, which unlike with the female photos did not improve fit [χ2 = 0.78, df = 1, *p* = 0.38, n.s.; see supplement for model parameters]. We also checked if these results depended on the photoset the monkeys viewed, and here found that this did impact results: inclusion of an interaction between expression and photoset improved model fit [χ2 = 22.63, df = 1, *p* < 0.0001]; post‐hoc tests showed that whereas monkeys looked longer at the threat than the neutral expression if they viewed photoset 1 [*p* = 0.0001], they looked longer at the neutral photo if they viewed photoset 2 [*p* = 0.0022]. Thus, in this data set there were differences in responses to male photos depending on the identity of the male photo presented to the subject monkeys.

We next calculated a *difference score* (Threat looking time–Neutral looking time) to examine overall age‐related changes to emotional stimuli and compare responses to female versus male photos, following prior work (Rosati, Arre, et al., 2018). This sort of difference score is commonly used in looking time research with human infants (Kominsky et al., 2022; McCrink & Wynn, 2004; Oakes, 2017; Olineck & Poulin‐Dubois, 2009; Spelke et al., 1992). Here, this score indexed how monkeys allocated their looking at the threat photo while accounting for overall looking to a neutral expression of the matched conspecific, such that more positive scores indicate greater relative interest in the negative threat photo. On average, we found that the difference scores were negative for the female photos (M = −0.42 ± SE = 0.11), indicating longer relative looking to the neutral photo, and positive for male photos (0.30 ± 17) indicating longer relative looking to the threat photo. To analyze this, we constructed a base LMM accounting for *photo set*, *outgroup* photos, and subject *identity* as a random factor; the full model including *photo type* (male or female photos), subject's *age*, subject's *sex*, and the interaction between *age X photo type* improved model fit [χ2 = 19.73, df = 4, *p* = 0.0006]. We then looked at each predictor sequentially. Inclusion of *photo type* (male or female photos) improved fit [χ2 = 11.82, df = 1, *p* = 0.0006]; monkeys indeed had more positive scores to the male photos. While the inclusion of *age* only trended to improve fit [χ2 = 2.90, df = 1, *p* = 0.09], the further inclusion of both *age* and *sex* improved fit compared to the model without individual subject characteristics [χ2 = 7.01, df = 2, *p* = 0.03]: male subjects tended to have lower scores than females, and scores generally become more positive (indicating greater relative attention to the threat photograph) with increasing age. Finally, we examined if there was any difference in this trajectory for male versus female photos by including the *age X photo sex* interaction; this did not further improve fit [χ2 = 0.90, df = 1, *p* = 0.34, n.s.; see Figure 2b and supplement for model parameters] indicating similar age‐related increases in attention to threat for both male and female stimuli as measured by the difference score.

### 3.2 Stability and longitudinal changes in socioemotional responses

We then examined patterns of cognition across the subset of animals that completed the socioemotional responses task in both years of data collection. First, we tested for repeatability of individual responses across the two years of testing, while also controlling for age and sex. We examined repeatability for each trial separately, as these trials involved different kinds of stimuli (e.g., neutral *vs.* threat, and female *vs.* male photos), and we also were interested in capturing long‐term repeatability in response to the same stimuli, as opposed to short‐term repeatability over different successive trials. We found significant long‐term repeatability for responses to female neutral photos, female threat photos, and male threat photos over the 1‐year period (see Table 2 for statistical reporting). Notably, these estimates were stronger for responses to the threat photos for both kinds of stimuli.

**Table 2:** Repeatability in the socioemotional responses task.

*Experimental stimuli*	*R*	*S.E.*	*N*	*C.I.*	*p‐value*
Female neutral expression^a^	0.178	0.080	150	0.026, 0.342	0.019
Female threat expression	0.277	0.079	150	0.117, 0.419	0.0004
Male neutral expression^a^	0.176	0.101	81	0.00, 0.393	0.077, n.s.
Male threat expression	0.356	0.098	81	0.171, 0.559	0.0008

*Note*: Long‐term repeatability (R) estimates for looking time responses from cognitive experiments conducted approximately one year apart for the same individuals; each analysis also accounted for age and sex.

^a^ Indicates models with singular fit indicating little variance between individuals.

We further used GLMMs to test how performance in year one predicted performance in year two in a more holistic fashion, examining the multiple test trials in tandem. Here, we used the subset of data where a given monkey completed the same trials in both years. We first constructed a base gamma GLMM model of year two looking times that included the same variables used in the prior analyses (e.g., accounting for subject's *identity*, *age* in year two, *sex*, the *photoset*, *outgroup*, and the interaction of *photo type* and *expression*). In the second model, we then further added *year one looking time* (e.g., for the same stimulus) to test if individual looking time performance in year one predicted looking in year two. This improved fit [χ2 = 8.806, df = 1, *p* = 0.003; see Figure 2c and supplement for model parameters]: above and beyond our original predictors, year one predicted year two performance. Overall, this provides converging evidence that individual variation in cognitive responses were stable across testing.

Finally, we used this same subset of monkeys to examine longitudinal (within‐individual) age changes in responses to emotional stimuli. Specifically, we used repeated measures correlations to assess common within‐individual associations (Bakdash & Marusich, 2017). For example, repeated measures correlations revealed a within‐individual steeper decline in looking time with age [age slope = −0.80, *r* = −0.30, *p* = 0.0001] compared to equivalent LMMs estimates [estimate = −0.17, SE = 0.03, *t* = −6.44, *p* < 0.0001; see Figure 2d]. We found similar patterns of exacerbated within‐individual age declines for the *female threat photo* [repeated measures correlation: age slope = −0.89, *r* = −0.39, *p* < 0.0001; LMM age estimate: −0.11, SE = 0.03, *t* = −4.46, *p* < 0.0001], the *male neutral photo* [repeated measures correlation: age slope = −0.62, *r* = −0.25, *p* = 0.024; LMM age estimate: −0.15, SE = 0.04, *t* = −4.20, *p* < 0.0001], and the *male threat photo,* although this final trial did not show a significant within‐individual age effect [repeated measures correlation: age slope = −0.46, *r* = −0.18, *p* = 0.10, n.s.; LMM age estimate: −0.12, SE = 0.04, *t* = −2.65, *p* = 0.01]. Overall, this indicates a stronger negative association between age and looking time at the intra‐individual (longitudinal) level, compared to the more moderate mean negative relationship between age and looking time across monkeys estimated from mixed models incorporating both longitudinal and cross‐sectional age estimates.

### 3.3 Individual variation in gaze following task

**Figure 3:**
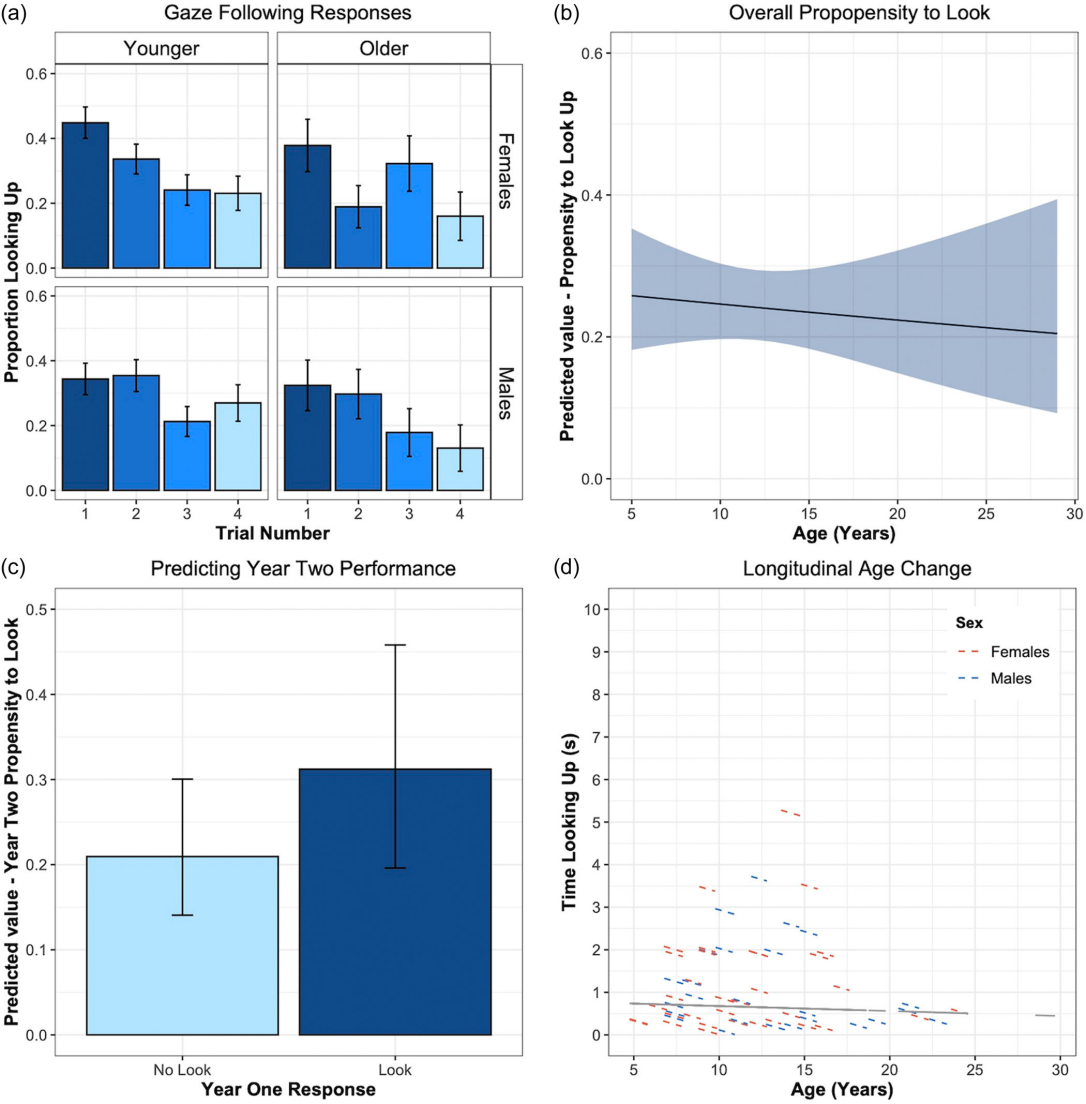
Individual variation in the gaze following task. (a) Propensity to follow gaze. Graph shows mean proportion of individuals looking up across the four trials, broken down by subjects' age and sex. Error bars indicate standard error. (b) Predicted propensity to follow gaze did not change with age. Estimated values are derived from generalized linear mixed‐effects models (GLMMs) of propensity to look up also accounting for subjects' identity, sex, age, and trial number. Ribbons indicate 95% confidence intervals. (c) Predicted performance over repeated testing. We tested if performance in year two was related to year one performance. There was no relationship between performance in year one and likelihood of looking up in year two for individuals who completed both years of testing. Estimated values are derived from GLMMS of propensity to look up accounting for subjects' identity, sex, age, and trial number. Ribbons indicate 95% confidence intervals. (d) Longitudinal changes in duration of looking responses. We used repeated measures correlations to isolate within‐individual associations in time looking up. The panel depicts responses to the first trial. The solid gray line indicates the (lack of) overall linear relationship between age and time spent looking up, whereas the dashed lines indicate the (lack of) within‐individual changes with age.

We first examined the proportion of monkeys following gaze across the 2 years of data. Overall, M = 38.6 ± SE = 2.9% of monkeys looked up on the first trial, 31.8 ± 2.8% of monkeys looked up on the second trial, 23.4 ± 2.8% of monkeys looked up on the third trial, and 22.2 ± 3.1% of monkeys looked up on the last trial (see Figure 3a for breakdown by age and sex). Following prior work (Rosati et al., 2016), we analyzed propensity to look across trials as our primary measure. We implemented a base model accounting for subject's *identity* as a random factor, and then in the full model included (1) *trial number* (as an ordinal factor, to test for habituation in responses over trials), (2) subject's *age*, (3) subject's *sex*; and (4) the interaction between *age* X *trial number*. This improved model fit compared to the base model [χ2 = 24.13, df = 4, *p* < 0.0001]. We then examined these factors sequentially. Inclusion of *trial number* improved fit compared to the base model [χ2 = 23.42, df = 1, *p* < 0.0001], revealing a significant effect of trial number: monkeys looked less across successive trials, indicating that they habituated to the repeated gazes of the actor. However, the subsequent additions of *age*, *sex*, and an *age X trial* interaction did not further improve fit [*p* > 0.5, n.s. in all cases; see Figure 3b and supplement for model parameters], indicating little variation in gaze following across individuals according to their demographic characteristics.

We next examined patterns of looking when the monkey did produce a look (i.e., only those trials where the monkey looked up). Overall, monkeys spent an average of 1.58 ± 0.09 s looking up when they produced a look, with similar durations of looking across trials. We confirmed this by analyzing duration of looking. Similar to the primary analyses of propensity to look, we implemented a base model accounting for subject's *identity* as a random factor, and then in the full model added *trial number, sex,* and *age* to subsequent models. There was no improvement in model fit for the full model [χ2 = 2.02, df = 3, *p* = 0.56, n.s.; see supplement for model parameters], and the individual inclusion of these factors also not improve model fit. Overall, this indicates that if monkeys did look up, the temporal characteristics of their looking responses were similar across trials, sexes, and cohorts.

### 3.4 Stability and longitudinal changes in gaze following responses

We next examined patterns of cognition across the subset of animals that completed the gaze following task in both years of data collection. We used the same approach to quantifying long‐term repeatibility as for the socioemotional responses task, here using binomial models, and again accounting for age and sex to model responses in each separate trial (note that these characterstics were removed in the analyses of trial 4 so that models could converge, given low sample size). This allowed us to test if individuals who looked up in the first year of testing also did so in the second year (rather than repeatibility over successive trials in the same year). In fact, we found significant repeatability for the first trial, but not the last three trials (although there was a trend for trial 4; see Table 3).

**Table 3:** Repeatability in the gaze following task.^a^

*Trial*	*R*	*S.E.*	*N*	*C.I.*	*p‐value*
Trial 1	0.157	0.077	102	0.00, 0.283	0.044
Trial 2	0.067	0.060	102	0.00, 0.213	0.209, n.s.
Trial 3	0.000	0.060	63	0.00, 0.205	1.0, n.s.
Trial 4^a^	0.243	0.345	37	0.00, 0.952	0.089, n.s.

*Note*. Long‐term repeatability (R) estimates for co‐orienting responses from experiments conducted approximately one year apart for the same individuals; each analysis also accounted for age and sex. All models had singular fit, indicating little variance between individuals.

^a^ Age and sex were not included in the model for trial 4, because separation of data prevented models from converging.

We further used GLMMs to see how performance in year one predicted year two in this subset of the monkeys. Specifically, we created a base model of year one gaze following responses (as a binary variable) accounting for the additional factors from our prior models: subject's *identity*, *age* in year two, *sex*, and *trial number*. We then added *year one response* (look or no look) for that trial in a second (full) model to test if individual performance was consistent over time. This did not improve fit [χ2 = 2.26, df = 1, *p* = 0.13, n.s.; see Figure 3c and see supplement for model parameters]. Overall, this aligns with the results from the repeatability analyses, indicating that an individual's gaze following responses were not particularly stable across repeated testing a year apart.

Finally, we used this same subset of monkeys to examine longitudinal (within‐individual) age changes in gaze following, using repeated measures correlations as in the socioemotional responses task. As repeated measure correlations can only use continuous data, for this analysis we used duration of looking up as a proxy for gaze following responses. Unlike in the socioemotional responses task, there was generally no significant effect of age using either approach. Repeated measures correlations were not different from 0 [e.g., for trial 1: repeated measures correlation: age slope = −0.12, *r* = −0.07, *p* = 0.50, n.s.; see Figure 3d], nor were LMMs estimates [for trial 1: age estimate = −0.012, SE = 0.020, *t* = −0.608, *p* = 0.54, n.s.]. This aligns with the results from the primary analyses, which also indicated no age effects, and further shows that observed declines were not detectable within‐individuals either.

### 3.5 Relationship between sociocognitive skills

**Figure 4:**
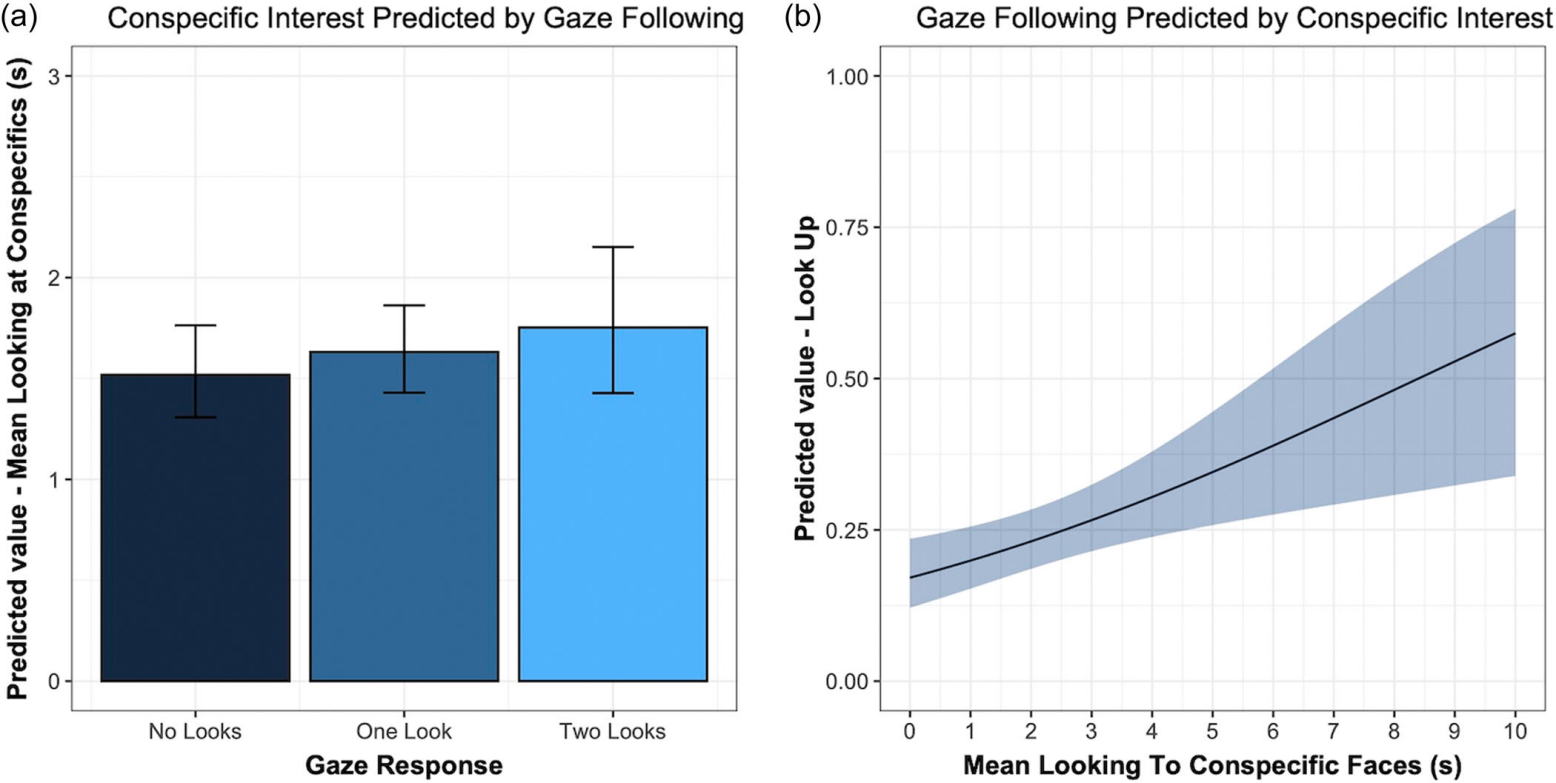
Inter‐task relationships. (a) Predicted performance in the socioemotional responses task from the gaze following task. Responses to conspecific photos for individuals who completed both tasks in a given year are depicted, broken down by their responses in the first two trials of the gaze following task (which all individuals completed; they therefore could have gaze followed on up to two trials). Gaze following did not significantly predict interest in the conspecific photographs. Estimated values are derived from linear mixed models also accounting for subjects' identity, sex, and age, and experimental variables. Error bars indicate 95% confidence intervals. (b) Predicted performance in gaze following task from the socioemotional responses task. Responses to gaze following demonstrations for individuals who completed both tasks in a given year are depicted, according to their average duration of looking at conspecific photos in the two trials of the socioemotional responses task (which all individuals completed). Individuals who looked longer at conspecific photos were more likely to look up in the gaze following task. Estimated values are derived from generalized linear mixed‐effects models of propensity to look up also accounting for subjects' identity, sex, age, and trial number. Ribbons indicate 95% confidence intervals.

We finally further used mixed models to test whether and how performance in one task was related to performance in the other in the same year of testing. First, we used gaze following to predict looking to conspecific faces in the socioemotional responses task, focused on the 151 monkeys who completed both tasks at least at one timepoint. We created a base gamma‐log link GLMM of looking times in the socioemotional task, mirroring the initial models that accounted for subject's *identity*, *age* at the time of the emotions task, *sex*, *photoset*, *outgroup photos*, and the interaction of *photo type* (female or male photos) and *expression* (neutral or threat). We then added *average gaze following responses* in a second (full) model, which did not improve model fit [χ2 = 1.31, df = 1, *p* = 0.25, n.s.; see Figure 4a and supplement for model parameters]. We then examined the reverse relationship by testing if attention to conspecific photos predicted gaze following. Specifically, we created a base binomial GLMM model of gaze following responses accounting for subject's *identity*, *age* at the time of the gaze task, *sex*, and *trial number*. We then added *average looking time* in a second (full) model, which here did improve fit [χ2 = 9.14, df = 1, *p* = 0.002; see Figure 4b and supplement for model parameters]. Overall, this shows that individual monkeys who showed more sustained interest in conspecific photographs were more likely to follow the gaze of a human actor, although propensity to engage in gaze following was a less‐reliable predictor of responses to the conspecific photos.

## 4 GENERAL DISCUSSION

We used a longitudinal approach to measure different aspects of social cognition in a large sample of rhesus monkeys across the adult lifespan at two time points. In the socioemotional responses task assessing attention to emotionally‐valanced versus matched neutral images, we found that monkeys exhibited general declines with age in social attention but also showed increases in relative attention to negative threat stimuli. These age‐related shifts were detectable also in longitudinal analyses. Our analysis showed that individual differences in performance in the task was repeatable and stable over repeated testing. In the gaze following task, we measured monkeys' co‐orienting response to an individual who looked up. Here, we did not find major age‐related differences either in the main analyses or in longitudinal‐only comparisons. While there was some limited evidence for repeatability, performance was not generally stable within individuals over time. Finally, analyses looking at the inter‐relationship of responses across these two tasks revealed that performance across these two distinct facets of social cognition was related: monkeys that looked longer at stimuli in the socioemotional responses task were more likely to co‐orient in the gaze following task (although gaze following did not similarly predict looking time responses). Overall, these results show that cognitive tasks implemented with free‐ranging animals can capture both age‐related changes and stable individual differences, but also that this can depend on the task in question.

Our findings of patterns of individual variation in the socioemotional responses task largely accorded with prior work using this task (Rosati, Arre, et al., 2018). Specifically, we found that the macaques generally exhibited declines with age in their attention to the conspecific photographs, but this was attenuated for responses to the negative threat expression in older age resulting in relative increases in attention to the threat photographs, with little variation by sex. This same general pattern was found in the analyses of looking time durations across trials, in comparisons of difference scores indexing an individual monkey's relative interest in the emotional threat stimuli, and finally in the longitudinal comparisons of the same monkey's responses over time. However, the current study did find more mixed responses to the male photographs compared to prior work (Rosati, Arre, et al., 2018), as here responses to these photographs varied across the two possible photo sets. Overall, these results align with prior findings showing that monkeys exhibit age‐related shifts in socioemotional attention that differ from patterns in humans, such that older monkeys show more relative interest in negative threat faces. Thus, while older macaques may retain a general interest in other's social interactions (Almeling et al., 2016), they also show an increasing negativity bias in several aspects of cognition and behavior (Machanda & Rosati, 2020). Notably, prior work with this task included a larger sample of monkeys (*n* = 337) including a cohort of juvenile individuals, whereas the current work shows that these same types of age‐related shifts in emotional biases are detectable in even in a sample comprised only of adults.

In the gaze following task, however, we found a different pattern of individual differences than seen in prior work (Rosati et al., 2016). While adults showed habituation over repeated successive trials as expected, there was no decline in propensity to follow gaze with increasing age, and adult females were not more responsive than adult males to the gaze cues. A lack of age‐related decline was found both not only in the cross‐sectional analyses comprising all data, but also in the longitudinal comparisons of individuals who were tested at two time points. As prior comparisons detecting sex differences and declines in propensity to follow gaze comprised both a much larger sample size (*n* = 481) and used a cross‐sectional approach with a larger cohort of infants and juveniles (Rosati et al., 2016), it is possible that these effects may require larger sample size to detect, or the inclusion of younger monkeys may heighten any such age‐related changes. However, studies of gaze following in this population similarly found age‐related declines in more comparable adult samples (Bettle & Rosati, 2019; Bettle & Rosati, 2022), suggesting that there may also be a true shift in gaze following patterns compared to prior work. Notably, the current data was collected after Hurricane Maria, a major natural disaster in the region, whereas those prior studies were conducted before this event. Accumulating evidence shows Hurriane Maria and other severe hurricanes have impacted some aspects of social behavior, life history outcomes, and biological aging in the Cayo Santiago population (Diaz et al., 2023; Morcillo et al., 2020; Testard et al., 2021; Watowich et al., 2022). Thus, one possibility is that patterns of co‐orienting also shifted in the Cayo Santiago rhesus macaques, such that older adults show higher attention or vigilance to gaze cues than did individuals before the hurricane. However, it is unclear why the hurricane might have impacted patterns of responding in the gaze following task but not in the socioemotional responses task, so this issue bears further study.

A novel aspect of the current work was the longitudinal design testing individuals at two time points a year apart, allowing us to examine repeatability and stability of cognitive performance in these tasks over a longer timescale. In fact, responses in the socioemotional responses task were stable and repeatable over this period, especially responses to the threat photos. While there is some increasing recent evidence for stability in some cognitive responses in animals (Cauchoix et al., 2018), most of this work has not focused on nonhuman primate species and has examined repeatability over shorter time frames than we did here. Our work therefore provides a first demonstration of such repeatability in a primate study using looking time methods over a fairly long (one‐year) time frame. This shows that even these quick cognitive assessments, taking only a few minutes, can capture at least some stable aspects of individual's cognitive responses. One possibility is that we found relatively high repeatability in the socioemotional responses task because it measures attention to biologically‐central stimuli: conspecific faces and emotional signals. Faces in general, and threats specifically, tend to quickly capture attention (Adolphs, 2008; Dal Monte et al., 2015; Hoffman et al., 2007; Lacreuse et al., 2013), and may therefore be less influenced by contextual factors that other psychological processes.

However, we found that in the gaze‐following task only responses on the first trial were repeatable, and there was no evidence that gaze following in the first year of testing overall was related to year two performance. This differing pattern might be related to the more complex nature of the task—which required not only attending to social agents, but also interpreting their social cues to co‐orient towards a distant target location in the external environment. Yet it is also possible that this different result is related to the smaller sample size assessed for repeatability on successive trials. Indeed, while some prior work with great apes has shown that individual variation in gaze following is stable over time (Bohn et al., 2023), that study involved more trials per individual than ours. As such, gaze following task may have provided less individual resolution compared to the looking time task. More generally, increasing attention to the issue of stable responses in cognition has revealed several cognitive and behavioral metrics that are not stable over time (Cauchoix et al., 2018; Mason et al., 2021; Rohrer & Ferkin, 2020; Soha et al., 2019), highlighting that repeatability in cognitive performance cannot necessarily be assumed.

Another major finding from the current study is that some aspects of performance across the two tasks was related: individual monkeys that showed longer looking at conspecific faces in the socioemotional responses task were more likely to follow the gaze of a human in the same year of testing, although we did not find support for the reverse relationship. This indicates that there may be some common underlying psychological trait that is reflected in performance in both tasks. Notably, prior work using a large set of tasks in primates has fairly consistently shown that there is often little commonality in responses to different social cognitive tasks. For example, work assessing a large cognitive task battery found that chimpanzees exhibit a “spatial” factor reflecting performance on tasks involving spatial cognition, but no specific factor reflect social cognitive performance across different tasks (Herrmann et al., 2010). Similarly, cross‐task relationships for great ape cognitive performance were lower when gaze following was included compared to when only non‐social tasks were assessed (Bohn et al., 2023). Some work has found no clear evidence that other cognitive tasks show shared variance in other nonhuman primate species (Völter et al., 2022). One possibility is that the interrelationships in the current tasks are in part capturing interest in and attention to social agents, a common aspect of both social cognitive tasks implemented here, and perhaps a more low‐level aspect of social behavior. In any case, this finding should be investigated further, given that the relationship we found was inconsistent.

One important issue to note is that the two tasks implemented here used quite different experimental measures. Both looking time and gaze following methods are commonly used with free‐ranging primates, given that both are appropriate for fast assessments of free‐ranging animals (Bettle & Rosati, 2019, 2021; Bettle & Rosati, 2022; De Petrillo & Rosati, 2019; Drayton & Santos, 2016; Drayton & Santos, 2017; Dubuc et al., 2016; Higham et al., 2011; Huang et al., 2024; Hughes & Santos, 2012; Marticorena et al., 2011; Martin & Santos, 2014; Martin & Santos, 2016; Rosati & Santos, 2017; Rosati et al., 2016; Rosati, Arre, et al., 2018; Teufel et al., 2010; Winters et al., 2015). Yet these tasks also have distinct methodological assumptions that should be carefully considered when interpreting results. For example, the socioemotional responses task used a preferential looking time task to measure monkey's interest in various stimuli, and increasing body of work shows that older monkeys generally look for shorter overall durations at a wide variety of both social and nonsocial stimuli (Bettle & Rosati, 2021; De Petrillo & Rosati, 2019; Huang et al., 2024). This highlights the importance of examining patterns of *relative* looking across conditions or stimuli, as we did here. In contrast, in the gaze following task the main measure is whether animals co‐orient with a demonstrator. Prior work has also shown that such responses do reflect co‐orienting in this population—in that they look in the same direction as the actor and show low baseline rates of looking up in relevant control conditions (Bettle & Rosati, 2019; Bettle & Rosati, 2022; Drayton & Santos, 2017; Rosati et al., 2016). However, we did not perform these kinds of control comparisons in the current study because gaze following in this species is well‐established (Bettle & Rosati, 2019; Drayton & Santos, 2017; Emery et al., 1997; Emery, 2000; Rosati & Santos, 2017; Rosati et al., 2016), and our main focus was on individual variation in gaze following. Moreover, both kinds of tasks might involve habituation over successive trials, but this means something different from a psychological perspective across the two tasks. Habituation is a core element of many looking time task designs, which are often aimed at either detecting dishabituation in response to a novel change or more generally preferential allocation of attention (Margoni, Surian, & Baillargeon, 2024; Winters et al., 2015). In contrast, habituation over successive trials of gaze‐following tasks is often taken as a marker of cognitive flexibility, as it indicates that animals control an adjust their responses rather than engaging in reflexive or automatic co‐orienting repeatedly to the same gaze cue even when there is no actual target to detect (Rosati & Hare, 2009; Rosati et al., 2016; Schloegl et al., 2007; Tomasello et al., 2001). Finally, it is important to note that while the socioemotional task used conspecific photographs, the gaze‐following task used a human actor. While previous research shows that macaques robustly follow the gaze of both humans and conspecifics, and appear to do so at similar rates (Ferrari et al., 2000; Rosati & Santos, 2017; Rosati et al., 2016; Teufel et al., 2010; Tomasello et al., 2001), there may be differences in how monkeys respond to some aspects of human versus conspecific social signals. Nonetheless, the fact that performance on these tasks were related in a given testing year suggests both are tapping into some shared social sensitivity.

Characterizing individual differences in cognition in the Cayo Santiago rhesus macaques allows for several new research questions moving forward. In particular, Cayo Santiago is a large population of monkeys living in naturalistic, freely‐breeding social groups—but are also very well‐habituated to human experimenters such that assessing cognitive performance using experimental techniques is possible as in the studies here. Moreover, demographics and social relationships in this population have been carefully documented over many generations (Ellis et al., 2019; Hernández‐Pacheco et al., 2016; Maestripieri & Hoffman, 2012; Newman et al., 2023; Rawlins & Kessler, 1986). In contrast, animals in many other primate populations where cognitive tests can be performed tend to live in smaller, human‐controlled groups. This means that studies of the Cayo Santiago macaques are uniquely well‐situated to connect cognitive traits to important biological outcomes such as health, longevity, and reproductive success. There is increasing evidence that social context impacts these kinds of outcomes in both humans and a variety of nonhuman animals (Dettmer & Chusyd, 2023; Lucas & Keller, 2020; Shively & Day, 2015; Snyder‐Mackler et al., 2020). For example, individuals who are socially well‐connected, have higher social status, or escape early life social adversity tend to have better health, longer lifespans, and greater fitness in several primate species (Morrison et al., 2021; Patterson et al., 2021; Silk et al., 2003; Tung et al., 2016). However, it is unclear whether or how underlying psychological propensities contribute to these kinds of effects.

The current work sets the stage for future work empirically testing these relationships between cognitive health, sociality and social status, and biological outcomes (Hernández‐Pacheco et al., 2023). Given the findings here, one possibility is that some aspects of cognitive performance may be more strongly related to fitness and health metrics than others. For example, socioemotional responses—which show clear and consistent patterns of individual variation—may be more predictive of health, reproduction, and longevity than gaze following, which was not as consistent within individuals over time. That is, socioemotional responses may reflect a more stable trait that could be related to long‐term biological outcomes and subject to the processes of natural selection. This may also be informative for understanding which cognitive abilities actually contribute to different metrics of social connectedness that are linked to aspects of fitness, such as longevity and reproductive success, in primates (Silk, 2007). Disentangling how different cognitive traits are linked to such real‐world outcomes is a critical next step for understanding the processes shaping the evolution of cognition across species.

## CONFLICT OF INTEREST STATEMENT

The authors declare no conflict of interest.

## ETHICS STATEMENT

All noninvasive behavioral tests reported in this paper were approved by the Institutional Animal Care and Use Committee of the University of Puerto Rico Medical Sciences Campus (protocol #A140116) and adhered to site guidelines for animal research. Research procedures adhered to the American Society of Primatologists Principles for the Ethical Treatment of Nonhuman Primates.

## Supporting information

Supporting information.

Supporting information.

Supporting information.

## Data Availability

Data is publicly available in Dryad Data Repository at: https://doi.org/10.5061/dryad.pzgmsbcw4
