# Supplementary material for: Individual differences in sociocognitive traits in semi‐free‐ranging rhesus monkeys (Macaca mulatta)
Source: Am J Primatol. 2024 Jul 4;87(2):e23660. doi: 10.1002/ajp.23660 (PMC11698962; doi:10.1002/ajp.23660)
Supplement: Supplementary file 1 — Supporting information. [file AJP-87-e23660-s003.docx]

**Supplementary Materials: Individual differences ­­in sociocognitive traits in**

**semi-free-ranging rhesus monkeys *(Macaca mulatta)***

A.A. Diaz, R. Hernández-Pacheco, & A.G. Rosati

**1. Supplemental methods**

As reported in the main text, in the socioemotional responses task we showed monkeys conspecific photographs with matched neutral versus threat expressions using an apparatus. Figure S1 depicts images of the apparatus and setup as well as examples of the conspecific photograph stimuli.


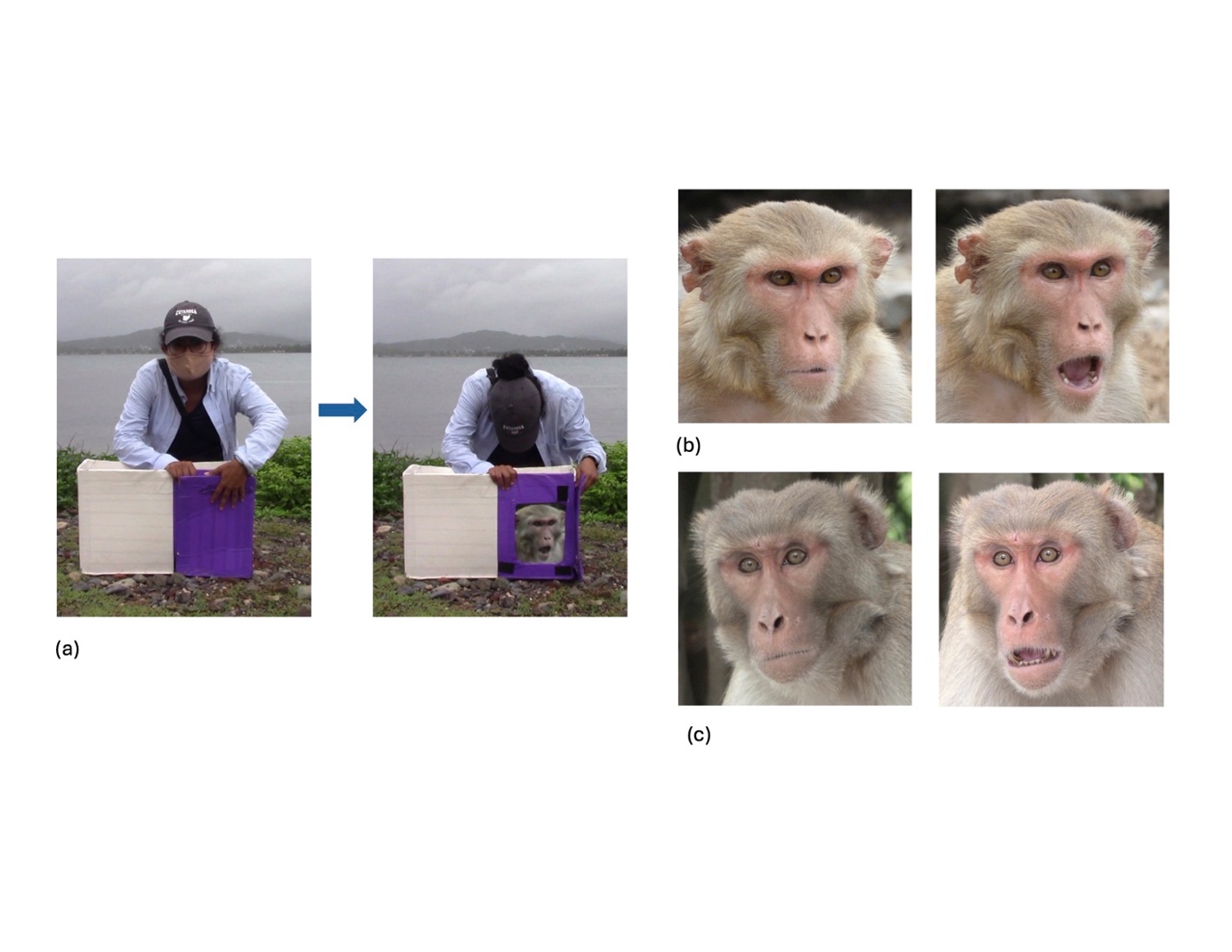


**Figure S1: Setup and stimuli for socioemotional responses task.** (a). We showed monkeys a photo of a conspecific by uncovering the window on an apparatus to reveal a conspecific monkey photograph. (b) In the first two trials, monkeys observed matched photos of a female conspecific producing first a neutral expression (left photo) and then the same individual producing a threat expression (right photo) (c) In the second two trials, monkeys observed matched photos of a male conspecific producing first a neutral expression (left photo) and then the same individual producing a threat expression (right photo).

**2. Supplemental Results**

*2.1 Socioemotional responses task: analyses of individual variation*

As reported in the main task, we first examined performance on all trials from monkeys who participated in this task in at least one of the testing years. Table S1 reports the parameter estimates for the full model from those analyses.

| **Predictor** | **Estimate** | **95% C.I.** | **S.E.** | ***t* value** | ***p* value** |
| --- | --- | --- | --- | --- | --- |
| Intercept | 1.720 | 1.205, 2.234 | 0.262 | 6.555 | < 0.0001 |
| Photoset (reference = 1) | -0.147 | -0.299, 0.004 | 0.077 | -1.901 | 0.0571 |
| Outgroup female photos (reference = no) | 0.059 | -0.342, 0.460 | 0.204 | 0.289 | = 0.772 |
| Outgroup male photos (reference = no) | -0.19 | -0.490, 0.103 | 0.151 | -1.276 | = 0.201 |
| Face stimuli (reference = female photos) | -0.328 | -0.477, -0.179 | 0.075 | -4.326 | < 0.0001 |
| Expression (reference = neutral) | -0.244 | -0.376, -0.112 | 0.067 | -3.621 | = 0.0002 |
| Age (in years) | -0.076 | -0.099, -0.054 | 0.011 | -6.778 | < 0.0001 |
| Sex (reference = female) | -0.007 | -0.243, 0.228 | 0.120 | -0.064 | = 0.948 |
| Face stimuli X Expression | 0.358 | 0.150, 0.565 | 0.105 | 3.386 | =0.0007 |

**Table S1: Predictors of looking to all photographs in the socioemotional responses task.** Parameters are from the full model (model 4), but the best fit model did not include sex; reference level for the predictors are noted in the table as relevant.

As reported in the main text, we then examined predictors of looking to the female photographs using the whole dataset. Table S2 reports the parameter estimates for the full model (which was the best fit) from those analyses.

| **Predictor** | **Estimate** | **95% C.I.** | **S.E.** | ***t* value** | ***p* value** |
| --- | --- | --- | --- | --- | --- |
| Intercept | 1.702 | 1.153, 2.250 | 0.279 | 6.079 | < 0.0001 |
| Photoset (reference = 1) | -0.168 | -0.341, 0.003 | 0.087 | -1.920 | = 0.0547 |
| Outgroup (reference = no) | 0.088 | -0.334, 0.512 | 0.216 | 0.410 | = 0.681 |
| Sex (reference = female) | 0.101 | -0.133, 0.335 | 0.119 | 0.846 | = 0.397 |
| Age (in years) | -0.098 | -0.124, -0.071 | 0.013 | -7.265 | < 0.0001 |
| Expression (reference = neutral) | -0.547 | -0.867, -0.227 | 0.163 | -3.349 | = 0.0008 |
| Age X Expression | 0.026 | 0.001, 0.050 | 0.012 | 2.116 | = 0.034 |

**Table S2: Predictors of looking to female photographs in the socioemotional responses task.** Parameters are from the full model (model 5: which was also the best-fit model); reference level for the predictors are noted in the table as relevant.

As reported in the main text, our second set of analyses for the socioemotional responses task examined predictors of looking to the male photographs using the whole dataset. Table S3 reports the parameter estimates for the full model from those analyses.

| **Predictor** | **Estimate** | **95% C.I.** | **S.E.** | ***t* value** | ***p* value** |
| --- | --- | --- | --- | --- | --- |
| Intercept | 1.444 | 0.817, 2.071 | 0.319 | 4.514 | < 0.0001 |
| Photoset (reference = 1) | -0.246 | -0.493, -0.000 | 0.125 | -1.960 | = 0.0498 |
| Outgroup (reference = no) | -0.195 | -0.602, 0.211 | 0.207 | -0.941 | = 0.346 |
| Sex (reference = female) | -0.069 | -0.509, 0.369 | 0.224 | -0.311 | = 0.755 |
| Age (in years) | -0.070 | -0.104, -0.036 | 0.017 | -4.025 | < 0.0001 |
| Expression (reference = neutral) | -0.120 | -0.439, 0.1978 | 0.162 | -0.742 | = 0.458 |
| Age X Expression | 0.015 | -0.019, 0.050 | 0.017 | 0.884 | = 0.376 |

**Table S3: Predictors of looking to male photographs in the socioemotional responses task.** Parameters are from the full model (model 5; the best fit model 3 included age but not sex or age X expression); reference level for the predictors are noted in the table as relevant.

As reported in the main text, our third set of analyses for the socioemotional responses task examined predictors of the looking time difference score (Looking to Threat - Looking to Neutral Photograph) using the whole dataset. Table S4 reports the parameter estimates for the full model from those analyses.

| **Predictor** | **Estimate** | **95% C.I.** | **S.E.** | ***t* value** | ***p* value** |
| --- | --- | --- | --- | --- | --- |
| Intercept | -0.027 | -1.025, 0.970 | 0.509 | -0.053 | = 0.957 |
| Photoset (reference = 1) | -0.497 | -0.900, -0.094 | 0.205 | -2.420 | = 0.016 |
| Outgroup (reference = no) | -0.661 | -1.357, 0.034 | 0.355 | -1.862 | = 0.063 |
| Sex (reference = female) | -0.427 | -0.847, -0.008 | 0.214 | -1.998 | = 0.047 |
| Age (in years) | 0.053 | 0.001, 0.105 | 0.026 | 2.020 | = 0.044 |
| Face (reference = female photos) | 1.127 | 0.129, 2.124 | 0.509 | 2.214 | = 0.027 |
| Age X Face | -0.037 | -0.116, 0.040 | 0.040 | -0.945 | = 0.345 |

**Table S4: Predictors of difference scores in the socioemotional responses task.** Parameters are from the full model (model 5; the best fit model 4 included age and face, but not the age X face interaction); reference level for the predictors are noted in the table as relevant.

*2.2 Socioemotional responses task: stability across years*

As reported in the main text, we next used the socioemotional responses task to examine if there was stability in individual performance across year by examining if inclusion of Year 1 looking time as a predictor improved fit above and beyond the primary experimental predictors. These analyses used looking to both female and male photos (when such data was available). Table S5 reports the parameter estimates for the full model from those analyses.

| **Predictor** | **Estimate** | **95% C.I.** | **S.E.** | ***t* value** | ***p* value** |
| --- | --- | --- | --- | --- | --- |
| (Intercept) | 0.516 | -0.099, 1.132 | 0.314 | 1.6435 | = 0.10 |
| Photoset in Yr 2 (reference = 1) | -0.188 | -0.495, 0.117 | 0.156 | -1.208 | = 0.227 |
| Outgroup in Yr 2(reference = no) | 0.315 | -0.032, 0.662 | 0.177 | 1.7753 | = 0.076 |
| Sex (reference = female) | 0.092 | -0.211, 0.3971 | 0.155 | 0.5960 | = 0.551 |
| Age in Yr 2 (in years) | -0.052 | -0.083, -0.020 | 0.016 | -3.268 | = 0.001 |
| Face (reference = female photos) | -0.283 | -0.497, -0.069 | 0.109 | -2.599 | = 0.009 |
| Expression (reference = neutral) | -0.366 | -0.536, -0.196 | 0.086 | -4.216 | < 0.0001 |
| Looking Time in Yr1 | 0.064 | 0.021, 0.106 | 0.021 | 2.9528 | = 0.003 |
| Face X Expression | 0.613 | 0.329, 0.898 | 0.145 | 4.2229 | < 0.0001 |

**Table S5: Predicting year 2 performance from year 1 performance in the socioemotional responses task.** Parameters are from the full model (model 2, the best fit model); reference level for the predictors are noted in the table as relevant.

*2.3 Gaze following task: analyses of individual variation*

As reported in the main text, our first set of analyses for the gaze following task examined predictors of gaze following (whether or not the monkey ever looked). Table S5 reports the parameter estimates for the full model from those analyses.

| **Predictor** | **Estimate** | **95% C.I.** | **S.E.** | ***z* value** | ***p* value** |
| --- | --- | --- | --- | --- | --- |
| Intercept | 0.136 | -0.998, 1.270 | 0.578 | 0.235 | = 0.814 |
| Trial Number (1-4) | -0.470 | -0.888, -0.052 | 0.213 | -2.204 | = 0.027 |
| Age (in years) | -0.029 | -0.116, 0.057 | 0.044 | -0.669 | = 0.503 |
| Sex (reference = female) | -0.120 | -0.626, 0.386 | 0.258 | -0.464 | = 0.642 |
| Age X Trial number | 0.007 | -0.024, 0.039 | 0.016 | 0.483 | = 0.629 |

**Table S5: Predictors of looking up in the gaze following task.** Parameters are from the model including age, sex, and age X trial number (model 5; the best fit model 2 included only trial number); reference level for the predictors are noted in the table as relevant.

As reported in the main text, our second set of analyses for the gaze following task examined predictors of duration of gaze following for those individuals who did produce looks. Table S6 reports the parameter estimates for the full model from those analyses.

| **Predictor** | **Estimate** | **95% C.I.** | **S.E.** | ***t* value** | ***p* value** |
| --- | --- | --- | --- | --- | --- |
| Intercept | 0.479 | 0.036, 0.923 | 0.226 | 2.122 | = 0.034 |
| Trial number (1-4) | -0.028 | -0.118, 0.062 | 0.046 | -0.609 | = 0.542 |
| Age (in years) | -0.019 | -0.049, 0.010 | 0.015 | -1.272 | = 0.203 |
| Sex (reference = female) | -0.015 | -0.301, 0.270 | 0.145 | -0.105 | = 0.916 |

**Table S6: Predictors of looking duration in the gaze following task.** Parameters are from the model including sex and age (model 4; none of these factors improved fit compared to the base model); reference level for the predictors are noted in the table as relevant.

*2.4 Gaze following task: stability across years*

As reported in the main text, we next used the gaze following task to examine if there was stability in individual performance across year by examining if inclusion of Year 1 looking response as a predictor improved fit above and beyond the primary experimental predictors, for the subset of individuals who completed the task in both years. Table S7 reports the parameter estimates from those analyses.

| **Predictor** | **Estimate** | **95% C.I.** | **S.E.** | ***z* value** | ***p* value** |
| --- | --- | --- | --- | --- | --- |
| Intercept | -0.621 | -1.881, 0.638 | 0.643 | -0.966 | = 0.334 |
| Trial number (1-4) | -0.395 | -0.708, -0.083 | 0.159 | -2.481 | = 0.013 |
| Age in Yr 2 (in years) | -0.001 | -0.076, 0.074 | 0.038 | -0.038 | = 0.970 |
| Sex (reference = female) | 0.309 | -0.442, 1.061 | 0.383 | 0.807 | = 0.419 |
| Look Y/N in Year 1 | 0.537 | -0.144, 1.220 | 0.348 | 1.544 | = 0.122 |

**Table S7: Predicting year 2 performance from year 1 performance in the gaze following task.** Parameters are from the model including Year 1 performance (model 2; this did not improve fit compared to the base model); reference level for the predictors are noted in the table as relevant.

*2.5 Task interrelationships*

As reported in the main text, we next used data where a given monkey completed both tasks in the same year to test if responses in the gaze following task predicted responses in the emotions task, and vice versa. Table S8 reports the parameter estimates for predicting looking in the socioemotional responses task from gaze following.

| **Predictor** | **Estimate** | **95% C.I.** | **S.E.** | ***t* value** | ***p* value** |
| --- | --- | --- | --- | --- | --- |
| Intercept | 2.007 | 1.311, 2.702 | 0.354 | 5.658 | < 0.0001 |
| Photoset (reference = 1) | -0.276 | -0.466, -0.086 | 0.097 | -2.849 | = 0.004 |
| Outgroup female photos (reference = no) | -0.113 | -0.656, 0.430 | 0.277 | -0.408 | = 0.683 |
| Outgroup male photos (reference = no) | -0.345 | -0.711, 0.020 | 0.186 | -1.851 | = 0.064 |
| Face stimuli (reference = female photos) | -0.276 | -0.450, -0.102 | 0.088 | -3.118 | = 0.002 |
| Expression (reference = neutral) | -0.184 | -0.340, -0.029 | 0.079 | -2.325 | = 0.020 |
| Age (in years) | -0.075 | -0.100, -0.050 | 0.012 | -5.891 | < 0.0001 |
| Sex (reference = female) | -0.013 | -0.281, 0.254 | 0.136 | -0.100 | = 0.920 |
| Mean gaze following | 0.143 | -0.099, 0.385 | 0.123 | 1.156 | = 0.247 |
| Face X Expression | 0.333 | 0.091, 0.575 | 0.123 | 2.698 | = 0.007 |

**Table S8: Predicting socioemotional task performance from gaze following performance in the same year.** Parameters are from the model including average gaze following response (which did not improve model fit); reference level for the predictors are noted in the table as relevant.

We conducted the same basis analysis in the referees direction. Table S9 reports parameter estimates for predicting gaze following from looking time in the socioemotional responses task.

| **Predictor** | **Estimate** | **95% C.I.** | **S.E.** | ***z* value** | ***p* value** |
| --- | --- | --- | --- | --- | --- |
| Intercept | -0.812 | -1.792, 0.167 | 0.500 | -1.624 | = 0.104 |
| Sex (reference = female) | -0.323 | -0.836, 0.189 | 0.261 | -1.236 | = 0.216 |
| Age (in years) | 0.0291 | -0.027, 0.085 | 0.028 | 1.011 | = 0.312 |
| Trial number (1-4) | -0.423 | -0.593, -0.253 | 0.086 | -4.885 | < 0.0001 |
| Mean socioemotional looking time | 0.188 | 0.065, 0.310 | 0.062 | 3.0150 | = 0.0025 |

**Table S9: Predicting gaze following performance from socioemotional task performance in the same year.** Parameters are from the model including average looking time (model 2; the best fit model); reference level for the predictors are noted in the table as relevant.

**3. Supplemental Movie Captions**

*Video S1: Socioemotional responses task demonstration and example monkey looking responses.* The first two videos show an example experimental demonstration of the procedure. In Video 1, the primary experimenter (the demonstrator) attracts the monkey’s attention to the apparatus, and then uncovers the window to reveal a conspecific neutral photograph. During the timed trials, she holds a position where she looks down with her hat covering her eyes. Video 2 shows the same procedure, here uncovering a conspecific threat photograph from the same individual. In between trials, the experimenter would move the photographs in the apparatus to set up the next trial. Videos 3, 4, and 5 show example coding clips illustrating monkey looking responses in the task. As illustrated in these videos, coding clips start a few seconds before the primary experimenter said “now” and did not contain any information about the trial type, such that coders could assess them blind to experimental variables. On the videos, the secondary experimenter (cameraperson) then says “stop” after at least 10s have passed; clips were always coded for exactly 10s from the moment the trial started to equate total trial duration across monkeys.

*Video S2: Gaze following task demonstration and example monkey responses.* In Video 1, the primary experimenter (the actor) attracts the monkey’s attention to her face, and then looks upwards. This demonstration includes a monkey’s response to the experimenter’s actions. In between trials, the experimenter would stand looking away from the monkey for at least 30s before starting the next trial using the same procedure. Videos 2, 3, and 4 show example coding clips illustrating monkey responses in the task. As illustrated in these videos, coding clips start a few second before the primary experimenter said “now” and do not contain any information about the monkey’s trial number, so that coders could assess them blind to experimental variables. On the videos, the secondary experimenter (cameraperson) then says “stop” after at least 10s have passed; clips were always coded for exactly 10s from the moment the trial started to equate total trial duration across monkeys.
